# Force Myography to Control Robotic Upper Extremity Prostheses: A Feasibility Study

**DOI:** 10.3389/fbioe.2016.00018

**Published:** 2016-03-08

**Authors:** Erina Cho, Richard Chen, Lukas-Karim Merhi, Zhen Xiao, Brittany Pousett, Carlo Menon

**Affiliations:** ^1^MENRVA Research Group, School of Engineering Science, Simon Fraser University, Burnaby, BC, Canada; ^2^Barber Prosthetics Clinic, Vancouver, BC, Canada

**Keywords:** force myography, force sensing resistors, classification, transradial amputee, residual limb, grip

## Abstract

Advancement in assistive technology has led to the commercial availability of multi-dexterous robotic prostheses for the upper extremity. The relatively low performance of the currently used techniques to detect the intention of the user to control such advanced robotic prostheses, however, limits their use. This article explores the use of force myography (FMG) as a potential alternative to the well-established surface electromyography. Specifically, the use of FMG to control different grips of a commercially available robotic hand, Bebionic3, is investigated. Four male transradially amputated subjects participated in the study, and a protocol was developed to assess the prediction accuracy of 11 grips. Different combinations of grips were examined, ranging from 6 up to 11 grips. The results indicate that it is possible to classify six primary grips important in activities of daily living using FMG with an accuracy of above 70% in the residual limb. Additional strategies to increase classification accuracy, such as using the available modes on the Bebionic3, allowed results to improve up to 88.83 and 89.00% for opposed thumb and non-opposed thumb modes, respectively.

## Introduction

The loss of a limb, regardless of the cause, has a significant negative impact on the individual. Prostheses are devices designed to mitigate this loss and have existed since the ancient Egyptian era. Today, the technology of prostheses has evolved considerably; in the case of upper extremity devices, robotic multi-dexterous hands, such as Otto Bock’s Michelangelo hand, Touch Bionics’ i-Limb, and Steeper Group’s Bebionic3 (Connolly, 2008; Medynski and Rattray, 2011; Belter et al., 2013), have been commercially available in the last decade.

However, despite recent technological advances, the overall rate of prostheses use in upper extremity amputees remain low (Biddiss and Chau, 2007) as the state-of-the-art is still far from effectively emulating the human hand and arm (Peerdeman et al., 2011). One of the problems is that, the increased complexity has introduced the new challenge of effectively controlling these devices (Østlie et al., 2012; Yang et al., 2014).

A large component of the difficulty of controlling these devices is due to their unreliability; misclassification of the user’s intentions frequently leads to unplanned movements (Biddiss et al., 2007). Although the conventional myoelectric control strategy involving two sEMG electrodes are sufficient for traditional myoelectric grippers involving only two configurations, opened and closed, the control of a more advanced terminal device requires a series of muscle cocontraction signaling, similar to Morse codes (Yang et al., 2014), making the user experience unintuitive and leading to human errors. One way the community has attempted to address this unintuitive control strategy is by including multiple sEMG electrodes to detect more subtle muscle activation profiles for various grips (Daley et al., 2010; Yang et al., 2014; Naik et al., 2015). Another approach was to modify the configuration of the sEMG electrodes placement (Fang and Liu, 2014). Meanwhile, others have focused more on the pattern recognition algorithm and a self-correcting system to sources of error in classification, such as inertia and force variation (Al-Timemy et al., 2013; Amsuss et al., 2014). However, one of the known limitations to classification accuracy and robustness is due to the sensors themselves. sEMG is prone to signal inconsistency due to interference from ambient noise, such as transmission from fluorescent lighting and televisions, changes in electrochemical signals due to sweat or humidity, electrode shifts as a result of limb movement, and signal cross-talking between adjacent muscles, which may make them unsuitable for prolonged use (Cram and Kasman, 1998; Oskoei et al., 2007; Castellini et al., 2014). Other recognized challenges include the adverse effects of limb position, weight, inertia, and force variation differences during the training on the pattern recognition performance (Cipriani, et al., 2011; Scheme, et al., 2010).

Other approaches, such as targeted muscle reinnervation (TMR) (Kuiken et al., 2009), electroneurography (ENG) (Cloutier and Yang, 2013), intracortical neural interfaces (Fang et al., 2015), and electrocorticography (ECoG) (Pistohl et al., 2012), all of which allow a more direct transmission of neural signals *via* surgical implants, have been explored. However, due to their invasive natures and costs, alternative non-invasive approaches have been sought. Examples of less invasive methods that have been explored include sonomyography, mechanomyography, electroencephalography, near infrared spectroscopy, magnetoencephalographic, and functional magnetic resonance imaging (Silva et al., 2005; Fang et al., 2015). Although all techniques have their own benefits and limitations, the main focus of this study was to investigate the use of one particular method, termed force myography (FMG) (Wininger et al., 2008).

Force myography, which is also referred to as residual kinetic imaging (RKI) (Phillips and Craelius, 2005) or muscle pressure mapping (MPM) (Radmand et al., 2014), is a technique involving the use of force sensitive resistors (FSRs) on the surface of the limb to detect the volumetric changes in the underlying musculotendinous complex. In a recent study (Ravindra and Castellini, 2014), researchers investigated the pros and cons of three types of non-invasive sensors, including sEMG, ultrasound, and FMG. In the scope of Ravindra and Castellini’s work, they concluded that FMG is the most promising of the three, as it has the potential to provide the highest accuracy in prediction, stability over time, wearability, and affordability of cost. In a different study, Li et al. (2012) investigated the use of FMG for classification and concluded that the use of FMG to decipher the user’s control intention was feasible. However, to the best of our knowledge, there have only been a small number of studies conducted on end-user subjects with transradial amputations.

This study compares the classification accuracy in the sound and residual limbs of four transradially amputated subjects and investigates whether the use of FMG is feasible. In addition, we demonstrated the control of a stand-alone commercially available prosthesis, Bebionic3, in real-time using the FMG technique (for demonstration video – see Supplementary Material).

## Materials and Methods

An experiment involving transradially amputated subjects in order to determine feasibility of the use of FMG to classify grip patterns was conducted. Forearm muscular deformation profiles were collected in both residual and sound limbs to compare classification accuracies for various grips.

### Hardware

**Figure 1:**
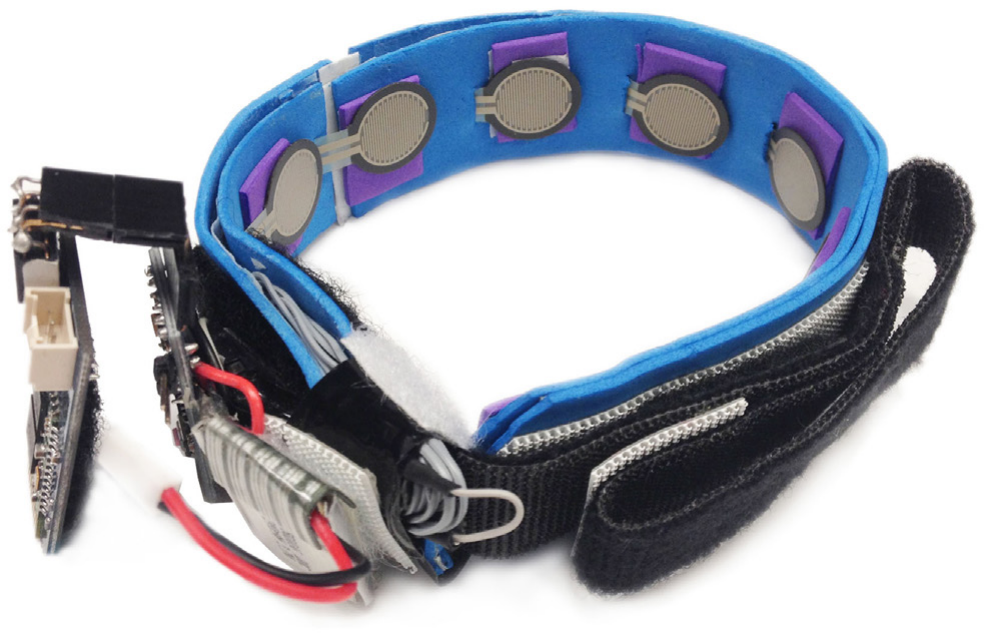
**FSR strap prototype developed at MENRVA Research Group**. The strap is 28.0 cm long and consists of eight embedded FSRs (FSR 402 from Interlink Electronics), which were evenly spaced on the strap’s inner surface. The strap itself is made of flexible chloroprene elastomeric (FloTex) foam with an adjustable Velcro to allow a customized fit user for various forearm circumferences.

To extract FMG signals, an FSR strap prototype (Xiao and Menon, 2014) developed by the MENRVA Research Group at Simon Fraser University was used and is shown in Figure 1. The strap is 28.0 cm long and consists of eight embedded FSRs (FSR 402 from Interlink Electronics), which were evenly spaced on the strap’s inner surface. The strap itself is made of flexible chloroprene elastomeric (FloTex) foam with an adjustable Velcro to allow a customized fit for various forearm circumferences.

**Figure 2:**
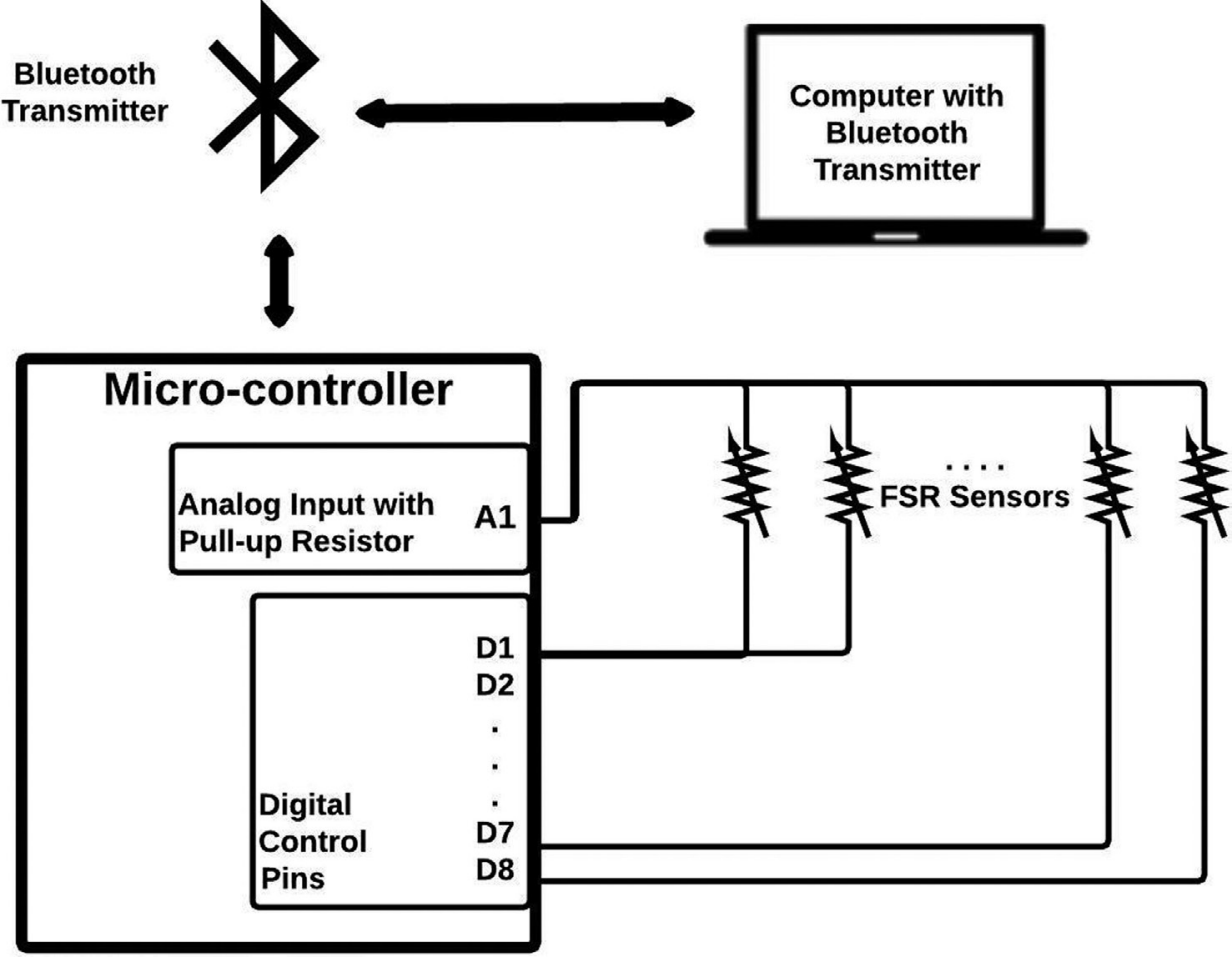
**Schematic for FMG signal extraction and transmission**. There are two terminals for each FSR. One terminal is connected to a common analog input pin of an Arduino ProMini micro-controller with an internal pull-up resistor of 37.5 kΩ, and the other to a digital control pin. The eight FSR signals are digitized sequentially using the micro-controller and transmitted *via* a Bluetooth module to a personal computer for data collection.

The signals from the FSRs were then extracted *via* a simple voltage divider circuit. There are two terminals in each FSR; one terminal is connected to a common analog input pin of an Arduino ProMini micro-controller with an internal pull-up resistor of 37.5 kΩ, and the other to a digital control pin and is schematically represented in Figure 2. The eight FSR signals were digitized sequentially using the micro-controller and transmitted *via* a Bluetooth module to a personal computer for data collection. The data collection software was developed in LabVIEW from National Instruments with a sampling rate of 10 Hz as proposed by Oliver et al. (2006). The sampling rate was selected in order to abide by the Nyquist criterion, where the sampling frequency must be twice the highest sampling frequency of movement in order to avoid the distortion of measured signals. Data in our study were collected in isometric conditions, and even in case of motion, since the frequency of human hand motion is typically <4.5 Hz (Xiong and Quek, 2006), 10 Hz is sufficient as the sampling rate for the purposes of the study.

### Protocol

**Figure 3:**
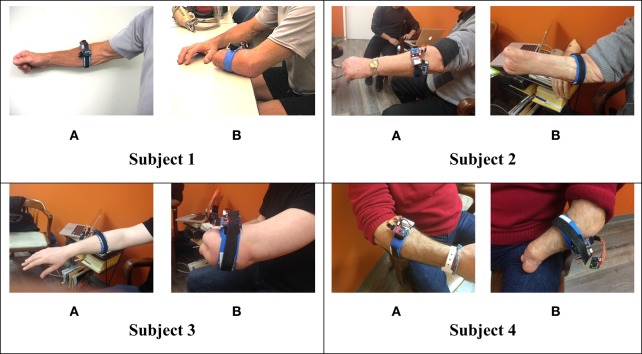
**FSR strap donned on the subjects’ (A) sound forearm (B) residual forearm**.

**Figure 4:**
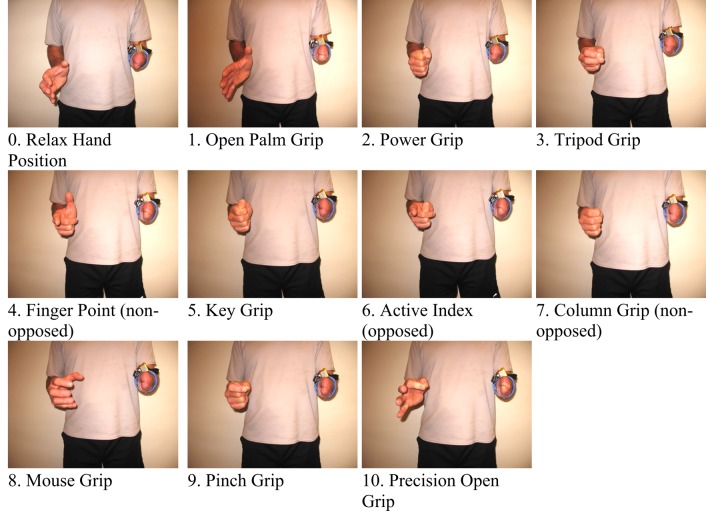
**The 11 grips tested for the study**.

In order to extract FMG data, an FSR strap was aligned to the bulk of the forearm, and donned first to the sound forearm and then the residual forearm as seen in Figure 3. Four grips, such as power grip, tripod grip, finger point (non-opposed), and key grip, have been identified as the most functional grips for activities of daily living by Peerdeman et al. (2011) and the most useful by Yang et al. (2014). In this study, these grips in addition to relaxed hand position and open palm are considered as the primary grips. Furthermore, five more grips available in the Bebionic3, were tested. In total, 11 grips were examined and are shown in Figure 4.

The subjects and the systems were trained by mirroring each grip in their residual limb with their sound limb as has done in previous experiments (Nielsen et al., 2011). The elbow was were flexed at 90° and each grip was held isometrically for a duration of three seconds per grip. The complete set was repeated five times, with 5 min of rest between each set as seen in Ravindra et al.’s previous work (Ravindra and Castellini, 2014). The grip sequences were kept the same for every set throughout the protocol to minimize confusion for the subjects.

### Subjects

Four transradially amputated male subjects were recruited for this study through Barber Prosthetics Clinic located in Vancouver, Canada. The clinical characteristics of each subject are described in Table 1. Although the sample size appears to be small at first, it is not, given the difficulty of recruiting transradially amputated individuals in this field (Atzori et al., 2014a,b). The average age of the subjects was 45 years old with a SD of ±17.2 years. All subjects provided written consent to testing after being informed of the testing procedure. The test procedure was approved by the Simon Fraser University Office of Research Ethics.

**Table 1:** **Clinical characteristics of subjects**.

Subject ID #	Sex	Age	Type of amputation	Time since amputation	Duration of myoelectric prosthesis use	Current device type
1	M	58	Acquired	35 years	2 years	Mechanical hook
2	M	64	Acquired	39 years	15 years	Myoelectric
3	M	21	Congenital	N/A	10 years	Myoelectric
4	M	36	Congenital	N/A	N/A	None

### Data Collection and Analysis

**Figure 5:**
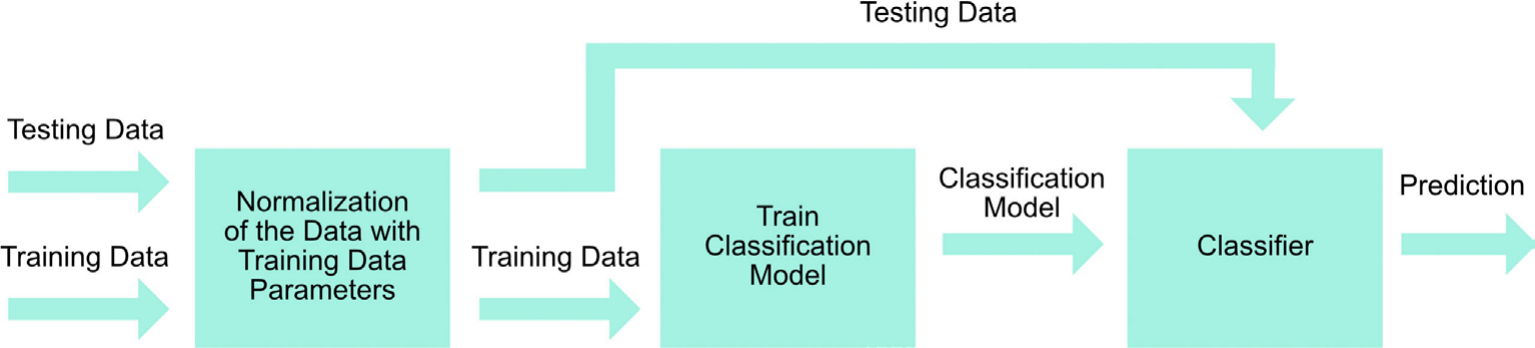
**Signal processing steps**.

The signal processing steps are described in Figure 5. Eleven grip gestures were recorded in a single trial, each grip gesture lasted 3 s (30 samples at 10 Hz), and a total of five trials were performed by each participant. The recorded FSR data were classified using the linear discriminant analysis (LDA) provided by MATLAB software from MathWorks. LDA was chosen for this study because of its ease to apply it in real-time, and ability to achieve similar or better classification results than other more complex methods (Englehart and Hudgins, 2003; Scheme and Englehart, 2011; Zhang et al., 2013; Amsuss et al., 2014).

First, we investigated the primary six grips. Second, we analyzed the 11 grip patterns, which were then subsequently categorized into the available opposed thumb and non-opposed thumb modes, based on the available control strategy of the Bebionic3. Inter-trial cross-validation was performed on the five trials in which four trials were used for training and one trial was used for testing. This resulted in five individual accuracies for each trial that was used for testing. The average accuracy was then obtained to represent the overall accuracy for all five trials.

## Results

**Figure 6:**
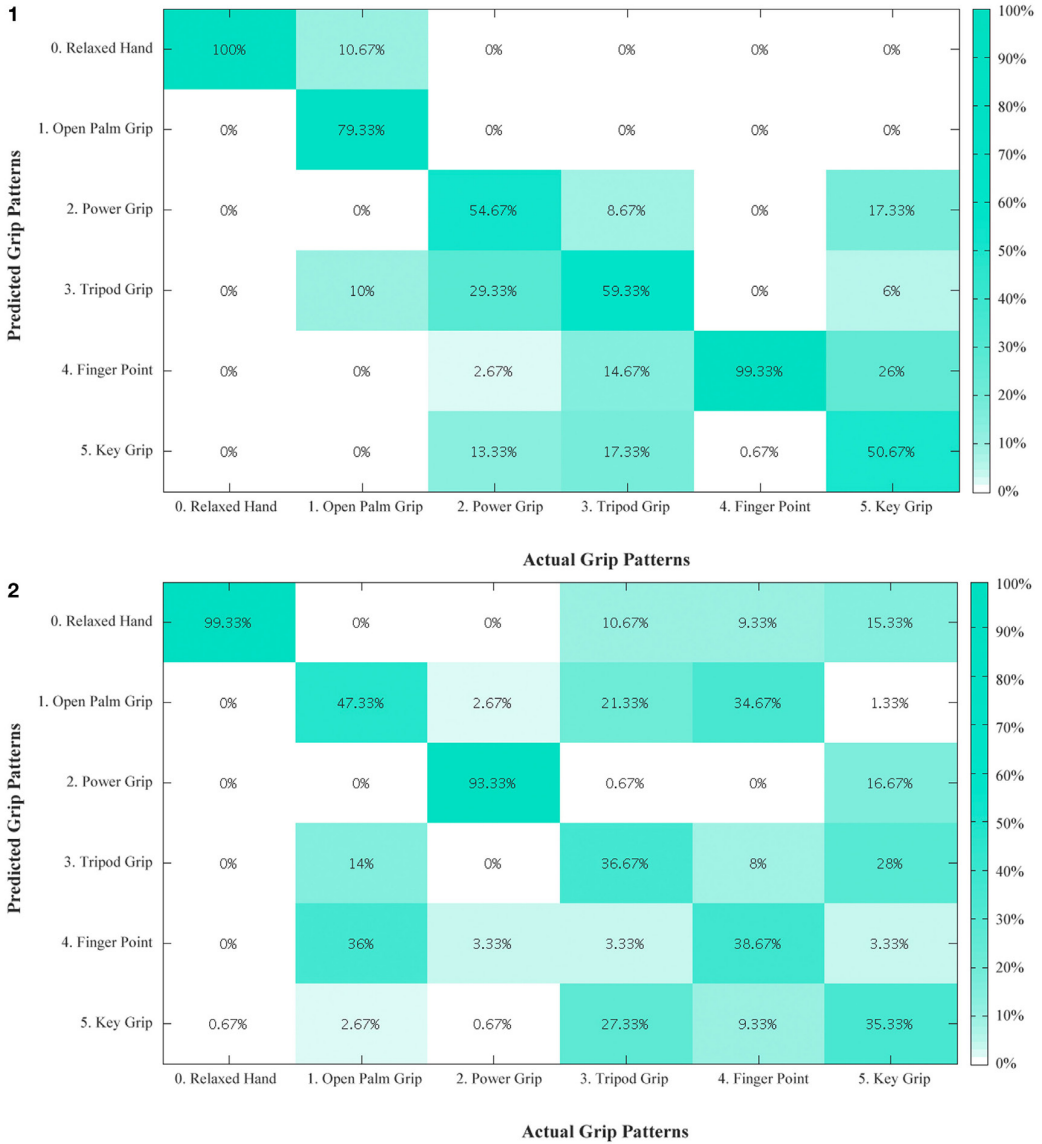

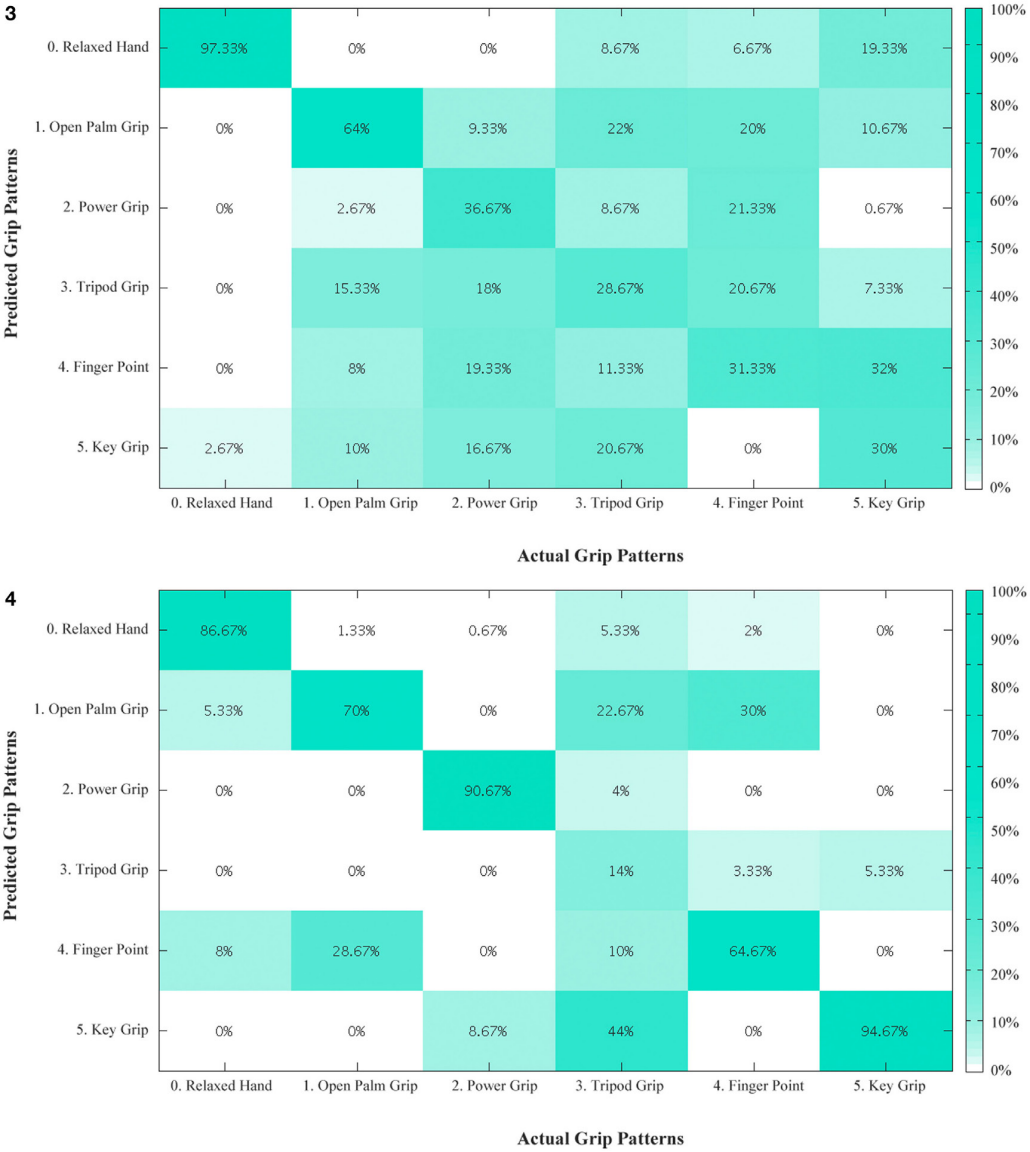
**Confusion matrix – primary grips for residual limb for subjects 1 to 4**. The diagonal entries represent the classification accuracy for the different grips. The off-diagonal entries represent inaccurate classifications. (1) For example, for subject 1, open palm grip in the second column is misclassified 10.67% of the time as relaxed hand and 10% of the time as tripod grip. (2) Confusion matrix – primary grips for residual limb for subject 2. (3) Confusion matrix – primary grips for residual limb for subject 3. (4) Confusion matrix – primary grips for residual limb for subject 4.

An overview of the classification accuracies for all the tested conditions are illustrated in Table 2. This analysis was performed for the primary grips identified as necessary for activities of daily living. With the six primary grips, an accuracy of up to 73.89% was achieved as shown in the confusion matrices in Figure 6 by taking the average of the diagonal. In all subjects, the classification accuracy of the sound limb was consistently higher than that of the residual limb. In addition, when all 11 grips were included, the classification accuracy decreased regardless of the individual. Among subjects, subject 1 and 4 appeared to have the best results, whereas subject 3 appeared to have the worst results throughout. When modes that were available on the Bebionic3 were taken into account, it was possible to increase the best classification accuracy from the six primary grips by approximately 18% in the opposed thumb mode for subject 2, and nearly 25% in the non-opposed thumb mode for subject 1. The confusion matrices for 11 grips, opposed thumb mode and non-opposed thumb mode of residual limb can be found in the Appendix.

**Table 2:** **Summary of classification accuracies**.

Subject ID#		1	2	3	4	Average
		Mean ± SD	Mean ± SD	Mean ± SD	Mean ± SD	Mean ± SD
11 Grips	Residual	42.60% ± 7.55%	47.45% ± 12.52%	21.58% ± 5.73%	55.29% ± 12.71%	41.73% ± 9.63%
	Sound	61.52% ± 6.52%	62.79% ± 15.16%	76.48% ± 14.77%	64.55% ± 12.19%	66.34% ± 11.94%
Primary grips (relaxed, open palm, power, tripod, finger point, key)	Residual	73.89% ± 6.92%	58.44% ± 12.69%	48.00% ± 15.82%	70.11% ± 10.73%	62.61% ± 11.54%
	Sound	67.00% ± 14.97%	69.67% ± 6.73%	94.67% ± 6.53%	83.33% ± 3.24%	78.67% ± 7.87%
Opposed thumb mode (relaxed, open palm, power, tripod)	Residual	82.17% ± 12.84%	81.67% ± 8.92%	67.00% ± 15.41%	88.83% ± 13.25%	79.92% ± 12.61%
	Sound	97.33% ± 2.53%	91.17% ± 10.86%	89.67% ± 11.14%	92.67% ± 9.78%	92.71% ± 8.58%
Non-opposed thumb mode (relaxed, open palm, finger point, key)	Residual	89.00% ± 9.55%	62.33% ± 17.57%	44.50% ± 9.23%	83.17% ± 7.67%	69.75% ± 11.01%
	Sound	84.00% ± 10.66%	96.50% ± 7.83%	100.00% ± 0.00%	99.33% ± 1.09%	94.96% ± 4.90%

## Discussion

According to the literature (Peerdeman et al., 2011; Yang et al., 2014), there are several grips that are considered more important in conducting activities of daily living than others. Variation in the results was seen between sound and residual limbs, subjects, and changes made to the original primary six grips.

It is not surprising that the sound limb consistently outperformed the residual limb. This is the case since the latter is missing a significant amount of anatomical features, such as bony landmarks that are insertion points for muscles, tendons, and ligaments alike, overall muscle volume due to atrophy from decreased use, and the ability for the residuum to produce distinct muscle deformation profiles. The combination of these factors negatively affects the FMG technique.

It is hypothesized that the discrepancy between subjects were due to a similar reason: musculature availability. As observed in Figure 3, subject 1 and 4, have a much longer residuum length than subject 3. Although subject 2 has a long residuum, there is significant atrophy of the muscles with the exception of the region slightly distal to his elbow, which could be one possible explanation for the lower classification accuracy.

The number of grips appeared to have an effect on the classification accuracy. With an increasing number of grips (from the 6 primary grips to 11 grips), the accuracy decreases for both limbs, indicating that it is more difficult to classify the user’s intentions. The decrease in classification accuracy was expected, as there are more grips with less distinct features that must be differentiated. The similarity between grips is observed in Figure 6.

To maximize the classification accuracy of the four primary grips, they can be separated into an opposed and non-opposed thumb configuration made available by Bebionic3’s hardware, while open palm and relaxed hand are included in both modes by default. The opposed mode includes the power and tripod grips while non-opposed mode includes finger point and key grips. Using this method, opposed thumb and non-opposed modes achieved an overall classification accuracy of up to 88.83 and 89.00%, respectively.

## Future Work

Future investigations should identify the optimal number of FSR sensors and examine other classification algorithms to improve classification accuracy in both static and dynamic states of the limb. A larger sample population with greater variety in residual limb lengths and resulting anatomical differences should also be accounted for, in order to assess the degree of robustness in classification of the FMG technique. In addition, a more realistic end-user condition should be established by using a system where the FSR sensors are embedded in a socket that is attached to the terminal prosthetic device. This should to be done to evaluate the effects of the dynamic mechanical environment in the socket, volumetric properties change within the residuum, and the weight on the comfort and function of the prosthesis.

## Conclusion

This article explored the use of FMG as a potential alternative to the well-established surface electromyography (sEMG). Specifically, the use of FMG to control different grips of a robotic hand was investigated. Four male transradially amputated subjects participated in the study. Different combinations of grips were examined ranging from 6 to 11 grips. The results indicate that it is possible to classify six primary grips important in activities of daily living using FMG with an accuracy of above 70% in the residual limb. Furthermore, results were made possible to be ameliorated up to 88.83 and 89% classification accuracy, when grips were subdivided into the opposed thumb and non-opposed thumb modes, respectively. Real-time control of a commercially available robotic prosthesis, Bebionic3, was also demonstrated. Further evaluation of FMG for the control of robotic upper extremity prosthesis will determine whether its optimal use is as an alternative or a synergist to the conventional EMG method.

## Author Contributions

EC: manuscript, data collection, and protocol design, RC: manuscript, data collection, data analysis, and protocol design, L-KM: manuscript, data collection, and data analysis, ZG: manuscript, data collection, and data analysis, BP: manuscript, CM: supervision and editing the manuscript.

## Conflict of Interest Statement

The authors declare that the research was conducted in the absence of any commercial or financial relationships that could be construed as a potential conflict of interest.

## Figures and Tables

**Figure A1:**
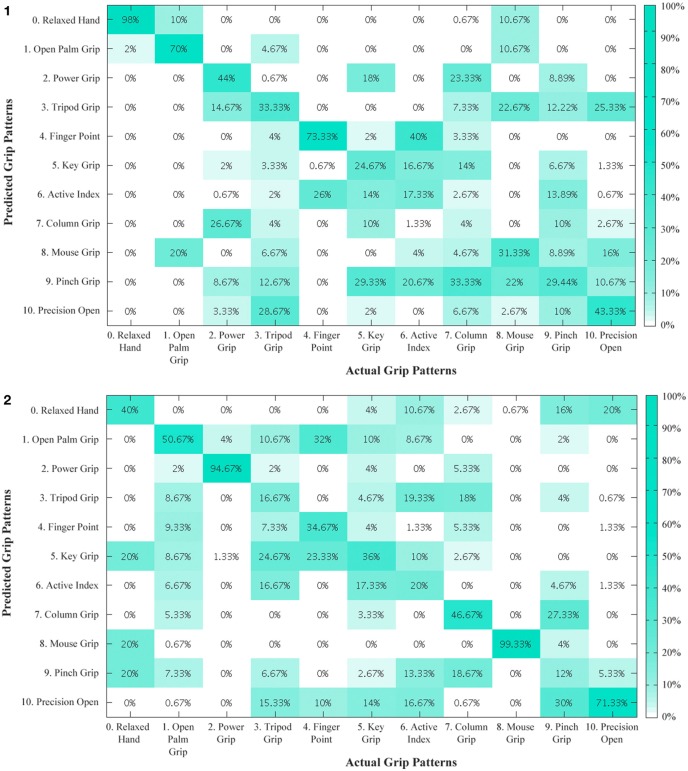

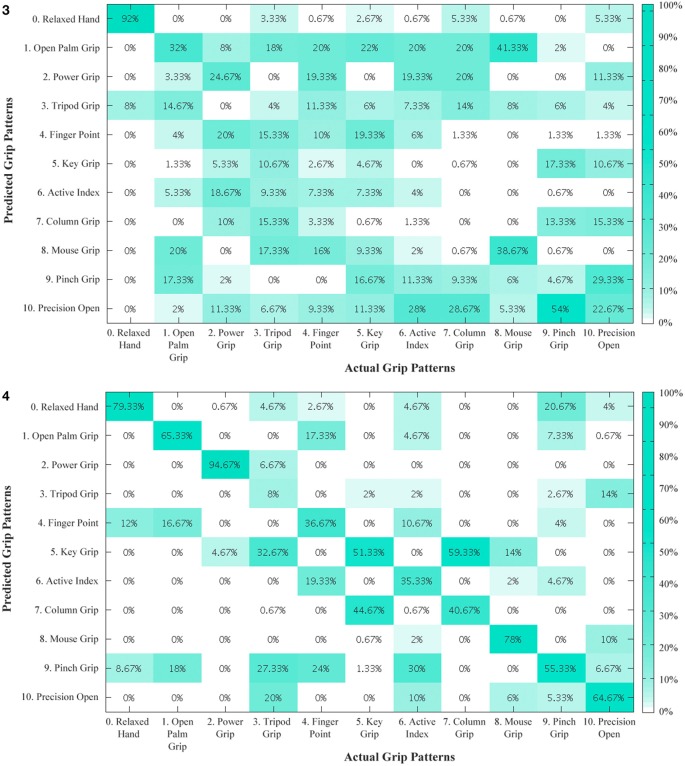
**Residual limb confusion matrix of all 11 grips for subjects 1 to 4**. The diagonal entries represent the classification accuracy for the different grips. The off-diagonal entries represent inaccurate classifications. For example, for subject 1, active index in the sixth column is misclassified 40% of the time as finger point. (2) Residual limb confusion matrix of all 11 grips for subject 2. (3) Residual limb confusion matrix of all 11 grips for subject 3. (4) Residual limb confusion matrix of all 11 grips for subject 4.

**Figure A2:**
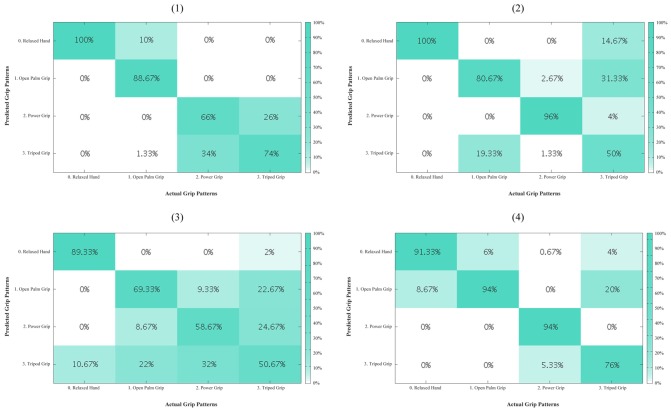
**Residual limb confusion matrices for opposed thumb mode for every subject (1–4)**. The diagonal entries represent the classification accuracy for the different grips. The off-diagonal entries represent inaccurate classifications. For example, for subject 1, tripod grip in the last column is misclassified 26% of the time as power grip.

**Figure A3:**
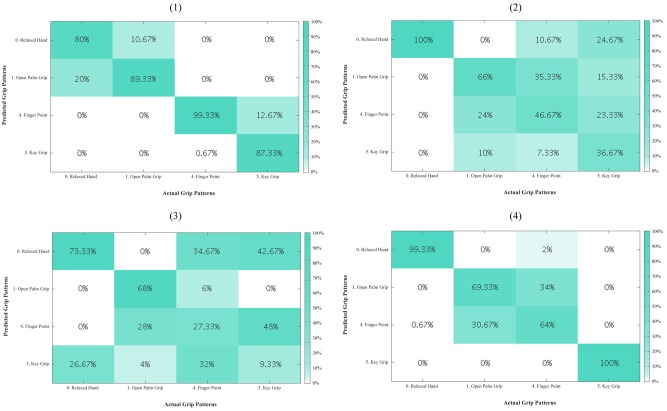
**Residual limb confusion matrices for non-opposed thumb mode for every subject (1–4)**. The diagonal entries represent the classification accuracy for the different grips. The off-diagonal entries represent inaccurate classifications. For example, for subject 1, key grip in the last column is misclassified 12.67% of the time as finger point.

## Supplementary Material

Video S1 **Demonstration video footage of the Bebionic3 being actuated using the FSR sensor strap**. The protocol was repeated for three grips: 1. Open Palm Grip; 2. Force Grip; 3. Tripod Grip in the opposed thumb configuration to show control of the Bebionic3. The video shows the Bebionic3 performing the grips intended by the subject, where the FSR strap is donned on the residual limb and the sound limb is used to mirror the intention of the residual limb. Additional grip patterns are not shown due to the limitations of the available hardware. **Hardware Setup for Demonstration Video** To demonstrate the feasibility of controlling a robotic prosthesis, the commercially available robotic multi-dexterous hand, Bebionic3 by Steeper Group, was used. To interface with this hand, an additional circuitry was developed to replicate the pre-existing sEMG signals recognized by most robotic prostheses. The signal produced by this circuitry replicates an open and close signal of an sEMG as well as an open–open signal to cycle through available grips. Live feedback was provided to the subject with a graphical user interface (GUI) to confirm whether the intended grip was detected properly. The GUI was developed in LabVIEW to perform real-time classification in addition to transmitting the classification results as commands (open, close, or open–open signals) to the Bebionic3 via Bluetooth. The additional circuitry consists of an Arduino ProMini microcontroller and two bipolar junction transistors (BJT) communicating with the GUI via Bluetooth. The BJT acts as a switch, one for each sEMG signal port, to create high and low impedances driven by two digital outputs from the Arduino ProMini. The FSR strap transmits sensor values via Bluetooth to LabView where the signal is processed and then classified. The classified grip command is then sent to the Arduino BJT circuit to open, close, or switch grips. The Bebionic3 has two thumb configurations, opposed and non-opposed, which is passively set in position by the sound limb. Each thumb configuration has two modes that can be cycled through with a hardware button found on the back of the Bebionic3. Each mode contains a primary and secondary grip, which can be cycled through with an sEMG open–open signal. Due to the limitations of the open–open signal, only two grips in addition to open palm may be selected actively.
